# Chondroblastoma associated with aneurysmal cyst of the navicular bone: a case report

**DOI:** 10.1186/1477-7819-11-50

**Published:** 2013-02-28

**Authors:** Zhenhua Fang, Ming Chen

**Affiliations:** 1 Department of Orthopedics, PuAi Hospital, affiliated to TongJi medical college, HuaZhong University of Science and Technology, Hanzheng Jie No. 473, Qiaokou District, 430033, Wuhan, China

**Keywords:** Chondroblastoma, Aneurysmal bone cyst, Navicular, Surgery

## Abstract

Chondroblastoma is a rare tumor. It is usually benign; however, it can have an aggressive course before or after operative treatment, even resulting in pulmonary metastases. The foot is a rare location for chondroblastoma, and to our knowledge, chondroblastoma occurring in the navicular bone has not been reported previously in the English literature. We describe a case of navicular chondroblastoma case associated with an aneurysmal bone cyst. Treatment consisted of aggressive curettage, phenolization, and bone allograft. The patient was able to resume normal activities after treatment, and there was no recurrence of the chondroblastoma during a follow-up of 3 years.

## Background

Chondroblastoma was first identified by Jaffe and Lichtenstein in 1942 [1]. It is a rare and usually benign bone tumor, which accounts for approximately 1% of all benign bone tumors [2]. There is a male preponderance, and there is a relatively higher frequency of chondroblastoma in the epiphyses of the long bones in adolescents. The two most common tumor locations are the proximal humerus and proximal tibia, with a male:female ratio of 2:1 [2-4]. Generally, the foot is a rare location for chondroblastoma, and approximately 20% of foot chondroblastomas occur in the calcaneus and talus bones [5]. In addition, there have been a few reported cases of chondroblastoma occurring on the cuboid bones [3,6,7]. To our knowledge, chondroblastoma of the navicular bone has not been reported previously in the English literature.

We report a case of and successful management of a navicular chondroblastoma associated with an aneurysmal bone cyst.

## Case presentation

A 24-year-old woman presented to the local clinic with pain in her left foot. She denied having any recent fevers, and she did not have any history of foot or ankle trauma. The rest of her medical history was not significant. The patient was initially treated conservatively with medication and rest. However, the pain gradually worsened, and began to be apparent even at rest. At this stage (about 6 months after her initial presentation), the patient was referred to our clinic for further treatment.

During the physical examination, the patient reported tenderness in the region of the navicular bone in her left foot, but there was no palpable mass in this area, and the temperature of the skin over the navicular bone was normal. The patient was unable to bear weight on her left foot, and the motor function of the foot was difficult to assess because of the pain.

**Figure 1:**
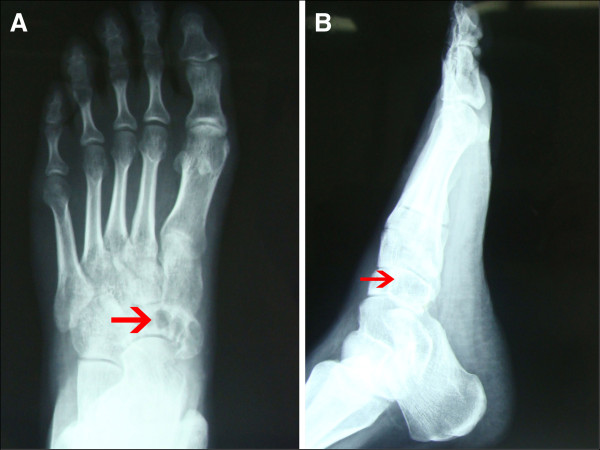
**Radiographs of the patient’s left foot.** (**A**) Anteroposterior view shows an expansile lesion of the navicular bone and (**B**) Lateral view shows thinning of the navicular cortex.

**Figure 2:**
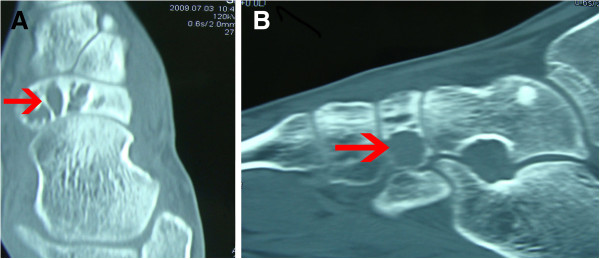
**Computed tomography image of the patient’s left foot.** (**A**, **B**) The cortex of the navicular seemed to be intact, with little intralesional matrix visible.

Anteroposterior and lateral radiographs of the left foot were taken, which showed an expansile lesion of the navicular bone without periosteal reaction (Figure 1A,B). Computed tomography showed thinning of the cortex, but the talonavicular joint was normal (Figure 2A,B). A technetium-99 m bone scan was also performed, which showed increased uptake of the tracer isolated to the left navicular. Based on the imaging data, the differential diagnosis included giant-cell tumor, aneurysmal bone cyst, and chondroblastoma.

A biopsy was taken from the lesion. An 18-gauge needle was used to puncture the lesion, and blood was aspirated easily from the lesion. Baseline chest radiography was also performed at this point. The result is that the blood in the lesion. Our result also based on these results).

**Figure 3:**
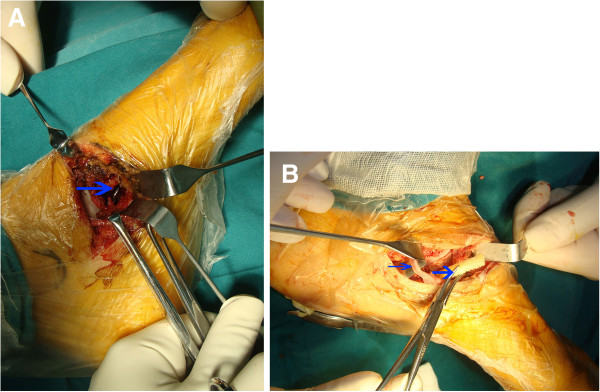
**Interoperative photographs.** (**A**) Lesion after curettage; (**B**) the allograft filled the cavity of the navicular bone (broad arrow), and the endpoint of the anterior tibia tendon is intact (thin arrow).

The patient was scheduled to undergo surgery including curettage, phenolization, and allograft, 3 days after her initial presentation to us. An incision 50 mm in length was made over the left medial navicular bone, and dissection of the tissue under the periosteum was performed. The upper cortex layer of the navicular bone was easily penetrated with a curette. The endpoint of the anterior tibial tendon was well protected (Figure 3A). The lesion was filled with red membranous tissue and blood. The navicular bone was curetted, and the tumor specimen was immediately sent for examination of frozen sections. The preliminary findings confirmed a benign tumor, consistent with either an aneurysmal bone cyst or chondroblastoma.

**Figure 4:**
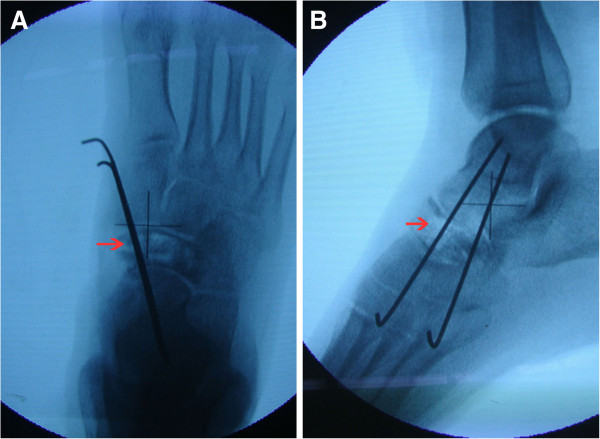
**Intraoperative fluoroscopy results.** (**A**) Anteroposterior view shows the two Kirschner wires used to fix the cortex; (**B**) lateral view shows normal joint anatomy.

A high-speed burr was then used to remove the entire lesion. This was followed by application of 89% phenol for 30 seconds, followed by neutralization with 100% alcohol for 1 minute, and then thorough irrigation with 1 litre of saline. Following the phenolization procedure, the allograft was placed into the cavity within the navicular bone (Figure 3B). Two 1.5 mm Kirschner wires were used to fix the cortex of the bone, and intraoperative fluoroscopy was performed to confirm the fixation (Figure 4A-B). A leg plaster was used to immobilize the leg.

**Figure 5:**
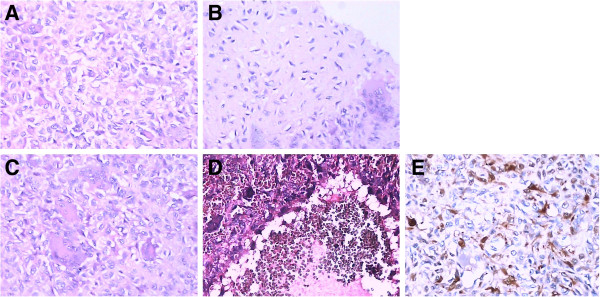
**Histological appearance of tumor.** (**A**) The chondroblasts had a clear cell membrane with pink cytoplasm and oval nucleus; (**B**) [ink cartilage-like matrix in the tumor; (**C**) Osteoclast-like giant cells were scattered throughout the tumor; (**D**) Cystic lesions concurrent with the aneurysmal bone cyst; (**E**) some of the chondroblasts in the cytoplasm and nucleus stained positively for S-100.

The tissue removed during surgery was sent for histological examination, and the final pathology diagnosis was navicular chondroblastoma associated with aneurysmal bone cyst. The tissue contained large polygonal chondroblast-like cells (Figure 5A) and pink cartilage-like matrix (Figure 5B), and large osteoclast-like giant cells were present in the lesion area (Figure 5C). There were also cystic lesions filled with blood, consistent with an aneurysmal bone cyst (Figure 5D). Some of the chondroblast-like cells in the cytoplasm and nucleus were positive for S-100 histology (Figure 5E). The pre-operative chest radiograph was interpreted as normal.

**Figure 6:**
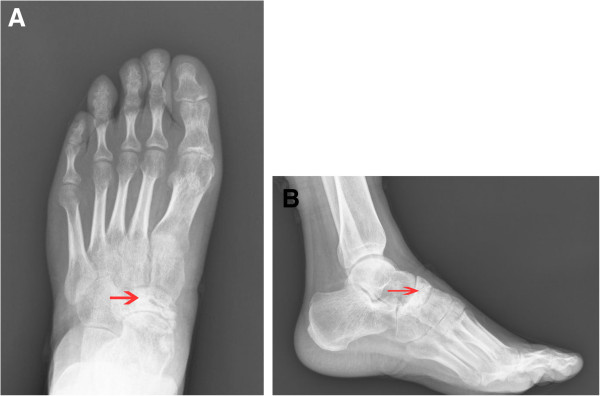
**A-B Post-operative radiographs.** Both anteroposterior and lateral views showed no recurrence of the tumor.

The plaster was removed from the patient’s leg after 6 weeks. The Kirchner wires were removed after the radiological confirmation of bony union. Post-operative radiographs at 3 months (Figure 6A-B) confirmed bony union.

The patient returned to her usual daily activities without pain. She had full range of motion of the mid-tarsal joints. She underwent follow-up including radiography at 3-month intervals for 1 year. Thereafter, radiographs were obtained biannually for 3 years, and yearly until she had completed 5 years of post-operative follow-up. At the 3-year follow-up, she was asymptomatic and there was no evidence of recurrence.

## Discussion

Chondroblastoma is a rare lesion. The majority (70%) of cases occur in the long bones, including the proximal humerus, proximal tibia. and femur [8]. The small bones are involved in only 10% of cases [5]. In this paper, we present a case of navicular chondroblastoma associated with an aneurysmal bone cyst. The successful treatment consisted of aggressive curettage, phenolization, and allograft reconstruction.

Pain was the main and presenting symptom in our case, which is compatible with previous reports [2,4]. Because of the frequent occurrence of this tumor near joints, effusion can be present in some patients. For this reason, chondroblastoma is often misdiagnosed as an inflammatory joint disease. Atalar *et al*. [4] reported a patient who had initially been misdiagnosed with a juvenile rheumatoid arthritis, and had undergone anti-inflammatory therapy for 1 year before the correct diagnosis was made. In our case, there was a six-month interval from the initial symptoms of pain to the suspicion of chondroblastoma, supporting the opinion that chondroblastoma is easily misdiagnosed [4].

In terms of the differential diagnosis, chondroblastoma should be distinguished from a number of other conditions. Giant-cell tumor is one possibility, but the tissue margins of chondroblastoma tend to be more clearly visible on imaging. [9] Chondroma, central chondrosarcoma and clear-cell chondrosarcoma can resemble chondroblastoma [9]. Similar to chondroblastoma, clear-cell chondrosarcoma often involves the ends of the long bones; however, it tends to occur in middle-aged patients, whereas chondroblastoma tends to occur in young patients [9]. Epiphyseal enchondroma has many points in common with chondroblastoma in terms of patient age and the skeletal distribution and location of the tumor [10]; however, histological examination can be used to distinguish between them [10]. 4) Inflammatory joint disease is another possibility [4]. Pain was the presenting symptom, Due to its more frequent occurrence near joints, chondroblastoma can present with joint pain and effusion. For this reason, it can be misdiagnosed as inflammatory joint disease.

Conventional radiographs can be helpful as an initial survey of a painful region. The involvement of the tarsal bones with cortical expansion and related erosive changes in the cortex is an indication of chondroblastoma. Usually, the lesion is located in secondary ossification centers [3]. Altalar *et al*. [4] reported that calcification in chondroblastoma was visible on radiographs in 59.4% of cases. A baseline chest radiograph should be obtained before diagnosis or surgery, and the diagnosis should be confirmed by biopsy. Annual chest radiographs should be performed for 5 years after treatment to monitor for pulmonary metastasis, which occurs in 1 to 3% of patients [3]. We should emphasize that the chondroblastoma might already display aggressive behavior at an early stage [2]. A bone scan may also be helpful to exclude other areas of skeletal involvement. Magnetic resonance imaging may be helpful to identify whether a soft-tissue mass is present.

Chondroblastoma is typically a slow-growing tumor [3,6,7]. Rarely, the tumor can grow rapidly, destroying the cortex and invading the soft tissues. However, Altalar *et al*. [4] reported that cortical destruction and soft-tissue invasion were seen in 13/32 patients with chondroblastoma. Our patient had no evidence of soft-tissue invasion. Secondary aneurysmal bone cysts have been reported by other authors [4,8], and this was also seen in our patient.

Atalar *et al*. [4] reported that local recurrence of chondroblastoma occurred in about 21.4% of their patients during the long-term follow-up. This recurrence rate is consistent with those in other studies [2,9]. Suneja *et al*. [2] reported that invasion of an open growth plate (physis) might imply the increase risk for local recurrence. However, Atalar *et al*. [4] analyzed potential risk factors for recurrence in 28 patients, and did not find that young age or the presence of an open physis to be associated with local recurrence. Nevertheless, controversy exists about what risk factors are associated with recurrence. The risk of recurrence has been reported as being higher in the hip and the pelvis, as a result of the difficult surgical access [5], but Suneja *et al*. and Atalar *et al*. [2,4] reported that there was no association between tumor location and recurrence. Generally, recurrence of chondroblastoma doe not seem to be associated with age, sex, tumor location, growth-plate status, or tumor activity [4].

In previous reports, it was suggested that surgical techniques can play a crucial role in chondroblastoma recurrence, as the actual removal of the tumor seems to be an important factor [2,4,8]. Simple curettage seems to result in a relatively higher recurrence rate compared with a standard curettage procedure [5]. A combination of curettage and cryosurgery was recommended by Schreuder *et al*. [11], which they reported as contributing to reducing the likelihood of recurrence. Lin *et al*. [8] reported that use of a burr could aid in extending the curettage, particularly in areas that are difficult to access. Atalar *et al*. [4] recommended cautery as a powerful tool in areas that were difficult to reach areas, as the heat generated could kill the tumor cells. A number of other surgical techniques are also available, including open and endoscopic curettage, curettage with fat implantation, marginal resection, resection with radiofrequency ablation, and osteochondral autograft transfer [8,12-14].

How to reconstruct the bone defect after surgery is another issue. Use of cement, bone graft, and other alternatives has been reported previously [6,14]. Sessions *et al*. [6] reported successful reconstruction using cement in cases of cuboid tumor. Reconstruction with cement can make immediate weight-bearing possible without risk of fracture, and can improve radiographic follow-up. However, the bone graft may resorb, making it difficult to differentiate the graft from a tumor recurrence [6]. The long-term effect of cement on the articular surface of the bone remains unknown, but it is possible that arthritis may develop, due to the non-anatomical construction of the cement [6]. At present, biological reconstruction using bone graft seems to be the best option, with good results reported by a number of authors [2,4,8]. The most common option for reconstruction after tumor excision is bone autograft harvested from iliac crest or fibula. The advantages of autograft include improved rate of graft incorporation and lack of immunogenic concerns [4,5]; however, it may need a longer operating time and can result in donor morbidity [15]. Use of allografot is an alternative option, which has been used for treatment of tumors in the calcaneus and talus bones [15,16]. However, fracture and non-union are common complications for allografts. There is also a risk of transmitting disease with allograft; however, with safety precautions, including donor selection and biological investigations, such risks are low [16]. In the present case, there were no complications related to the allograft.

## Conclusions

Generally, it is well accepted that aggressive curettage and bone graft are the preferred treatment for chondroblastoma [2,4,8]. In our case, our successful treatment consisted of an open biopsy followed by curettage, phenolization, and allograft. This case appears to be the first case of chondroblastoma occurring in the navicular bone. We hope this report will increase the awareness of this rare tumor occurring in the foot.

## Consent

Writing informed consent was obtained from the patient for publication of this case report and accompanying images.

## Competing interests

Both authors declare that they have no commercial interests in this report.
